# Evaluation of Machine Learning and Rules-Based Approaches for Predicting Antimicrobial Resistance Profiles in Gram-negative Bacilli from Whole Genome Sequence Data

**DOI:** 10.3389/fmicb.2016.01887

**Published:** 2016-11-28

**Authors:** Mitchell W. Pesesky, Tahir Hussain, Meghan Wallace, Sanket Patel, Saadia Andleeb, Carey-Ann D. Burnham, Gautam Dantas

**Affiliations:** ^1^Center for Genome Sciences and Systems Biology, Washington University School of MedicineSt. Louis, MO, USA; ^2^Atta ur Rahman School of Applied Biosciences, National University of Sciences and TechnologyIslamabad, Pakistan; ^3^Department of Pathology and Immunology, Washington University School of MedicineSt. Louis, MO, USA; ^4^Department of Pediatrics, Washington University School of MedicineSt. Louis, MO, USA; ^5^Department of Biomedical Engineering, Washington University in St. LouisSt. Louis, MO, USA; ^6^Department of Molecular Microbiology, Washington University School of MedicineSt. Louis, MO, USA

**Keywords:** antibiotics, resistance, diagnostics, *Enterobacteriaceae*, machine-learning, beta-lactam, aminoglycoside, tetracycline

## Abstract

The time-to-result for culture-based microorganism recovery and phenotypic antimicrobial susceptibility testing necessitates initial use of empiric (frequently broad-spectrum) antimicrobial therapy. If the empiric therapy is not optimal, this can lead to adverse patient outcomes and contribute to increasing antibiotic resistance in pathogens. New, more rapid technologies are emerging to meet this need. Many of these are based on identifying resistance genes, rather than directly assaying resistance phenotypes, and thus require interpretation to translate the genotype into treatment recommendations. These interpretations, like other parts of clinical diagnostic workflows, are likely to be increasingly automated in the future. We set out to evaluate the two major approaches that could be amenable to automation pipelines: rules-based methods and machine learning methods. The rules-based algorithm makes predictions based upon current, curated knowledge of *Enterobacteriaceae* resistance genes. The machine-learning algorithm predicts resistance and susceptibility based on a model built from a training set of variably resistant isolates. As our test set, we used whole genome sequence data from 78 clinical *Enterobacteriaceae* isolates, previously identified to represent a variety of phenotypes, from fully-susceptible to pan-resistant strains for the antibiotics tested. We tested three antibiotic resistance determinant databases for their utility in identifying the complete resistome for each isolate. The predictions of the rules-based and machine learning algorithms for these isolates were compared to results of phenotype-based diagnostics. The rules based and machine-learning predictions achieved agreement with standard-of-care phenotypic diagnostics of 89.0 and 90.3%, respectively, across twelve antibiotic agents from six major antibiotic classes. Several sources of disagreement between the algorithms were identified. Novel variants of known resistance factors and incomplete genome assembly confounded the rules-based algorithm, resulting in predictions based on gene family, rather than on knowledge of the specific variant found. Low-frequency resistance caused errors in the machine-learning algorithm because those genes were not seen or seen infrequently in the test set. We also identified an example of variability in the phenotype-based results that led to disagreement with both genotype-based methods. Genotype-based antimicrobial susceptibility testing shows great promise as a diagnostic tool, and we outline specific research goals to further refine this methodology.

## Introduction

The spread of antibiotic resistance has become an urgent threat to modern medicine's control over bacterial infections. In critically ill patients, it has been well established that the time taken to administer an appropriate antibiotic agent inversely correlates with improved patient outcomes (Kumar et al., 2006). Unfortunately, definitive *in vitro* antibiotic susceptibility testing (AST) results are not typically available until at least 2 days after specimens arrive in the clinical laboratory (Didelot et al., 2012), necessitating broad spectrum empiric antibiotic therapy.

Rapid antimicrobial resistance diagnostics could reduce the time to treatment with the optimal antibiotic therapy for the millions of patients infected with antibiotic resistant pathogens each year (CDC, 2013). Increasing pressure to improve antibiotic stewardship also favors the development of faster diagnostics (Caliendo et al., 2013; Barlam et al., 2016)—reducing the interval of diagnostic uncertainty will result in more judicious antimicrobial use. The growth rate of infectious agents imposes a limit on the speed with which phenotype-based AST can return results, meaning that a new diagnostic paradigm is needed to inform initial treatment.

Several diagnostic assays have emerged that rapidly identify antibiotic resistance based on genotypic rather than phenotypic information, including multiplex PCR and microarray assays designed to identify resistance-specific markers (Pulido et al., 2013; Kothari et al., 2014; Zumla et al., 2014). While these techniques can be successful at detecting resistance determinants mediated by specific enzymes, few of these methods can detect resistance mediated by target mutation, such as fluoroquinolone resistance (Pulido et al., 2013; Kothari et al., 2014; Zumla et al., 2014), and accuracy decreases as additional genes are assayed (Mancini et al., 2010).

Whole genome sequencing (WGS) has been proposed as a complementary method for identifying antibiotic resistance genes (Didelot et al., 2012; Bertelli and Greub, 2013; Zumla et al., 2014). As this approach interrogates the entire genome of each organism, WGS can identify the full set of known resistance factors in an organism including target-mediated resistance. WGS can also be used to identify new resistance gene variants, and can contribute to identifying entirely novel resistance gene families. Another advantage is that neither the DNA preparation nor the resistance gene identification steps increase in duration or cost as the number of antibiotics evaluated increases. Pathogen WGS is also being evaluated for other roles in clinical diagnostics, such as species identification (Wilson et al., 2014) and assessment of strain relatedness (Snitkin et al., 2012; Reuter et al., 2013), thereby affording the potential for a single test to be used for multiple purposes. Several authors have implemented WGS as a potential clinical diagnostic (Stoesser et al., 2013; Gordon et al., 2014; Hasman et al., 2014; Leopold et al., 2014), arguing that the time and cost of sequencing will decrease to acceptable levels in the near future. As further evidence of increasing interest in this technology, the United States Food and Drug Administration (FDA) has recently issued draft guidelines for diagnostic manufacturers seeking to develop new devices for WGS based infectious disease diagnostics (Sichtig, 2016).

Despite its potential benefits, any WGS-based diagnostic still has several hurdles to overcome before it can become a viable alternative to *in vitro* phenotypic tests. First, it is currently slower and more expensive than traditional phenotypic susceptibility testing. This limitation is likely to diminish over time, as innovations in sequencing methodologies are continually decreasing cost and assay duration (Feng et al., 2015; Wetterstrand, 2016). Second, the genes relevant to antibiotic resistance must be identified during the analysis phase, rather than being directly selected by the technique. This increases analysis time and introduces the potential that sequencing or alignment errors introduce inaccuracies in resistance gene identification.

Previous implementations of genotype-based diagnostics have focused on identifying antibiotic resistance determinants, without optimizing interpretation of the organism susceptibility profile (Pulido et al., 2013; Stoesser et al., 2013; Gordon et al., 2014; Hasman et al., 2014; Kothari et al., 2014; Zumla et al., 2014). Simple gene identification can suggest what antibiotics an infection may be resistant to, but they are inconclusive about what treatments will indeed be active. The emergence of transferrable resistance to last-resort drugs like colistin (Liu et al., 2016) and the carbapenems (Yigit et al., 2001), both in the context of already multidrug-resistant pathogens, virtually guarantees the spread of nearly pan-resistant isolates. Accurate identification of any antibiotic susceptibilities these isolates may still have is especially important. A true genotype-based antibiotic susceptibility prediction (GBASP) algorithm that could more rapidly provide all of the information given by current phenotypic AST methods would need to be able to provide this critical susceptibility information.

Providing susceptibility information that can directly inform treatment recommendations requires an additional layer of interpretation for GBASP than is found in current implementations of genotype-based diagnostics. It is critical to minimize errors in this interpretation step, as the results of this step will directly affect treatment decisions. The presence or absence of specific resistance genes must be associated with resistance (and susceptibility) to particular antibiotics, and then the resistance profiles for all genes in a particular isolate must be added together to provide the predicted susceptibility profile for that organism. The case of the β-lactamases in the *Enterobacteriaceae* highlights the potential difficulties of this interpretation. Single *Enterobacteriaceae* isolates may carry multiple β-lactamase genes, and the resistance provided by each of these resistance genes against the diverse class of β-lactam drugs (i.e., penicillins, cephalosporins, cephamycins, and carbapenems) is determined not just by the family to which those genes belong, but in some cases by specific amino acid substitutions (Bush and Jacoby, 2010).

In this study we evaluated multiple analytical approaches to interpreting WGS GBASP as an alternative to phenotypic AST. We first compared the performance of three curated antibiotic resistance sequence databases in identifying the genes important for resistance. Next, for the critical step of translating resistance gene identifications into resistance and susceptibility predictions, we compared a rules-based algorithm to a machine learning approach. In general, rules-based algorithms should be equally successful whether they are applied to a small or large set of pathogens and errors should be relatively easy to detect and fix, but they can only account for known factors that contribute to resistance. Machine learning algorithms should be able to deduce patterns from gene data regardless of whether those patterns are already known to the field, but they require large training sets to be effective and errors may be difficult to interpret. We therefore chose to implement both algorithms and compare their results. Our rules-based algorithm used a hard-coded resistance profile for each resistance gene identified, based on existing curated knowledge of resistance in the *Enterobacteriaceae*, while our machine learning approach treats all resistance genes equally and makes predictions based on algorithmically-deduced patterns in the data.

We applied this pipeline to predict the resistance profiles of 78 previously characterized genome-sequenced *Enterobacteriaceae* isolates (Pesesky et al., 2015) to 12 antibiotics. To best evaluate the predicted profiles as a clinical metric, we compared the results to the categorical interpretation of *in vitro* susceptibility testing determined by Kirby Bauer Disk Diffusion (CLSI, 2015), the method currently employed by the clinical microbiology laboratory of Barnes Jewish Hospital, St. Louis, MO. For each antibiotic class, we then assessed the strengths and weaknesses of each predictive algorithm. Our goal was to determine the current most effective implementation of automated interpretation for GBASP, how close that implementation came to standards necessary for clinical use, and to identify specific knowledge gaps needed to improve future iterations of this diagnostic approach.

## Methods and materials

### Sample selection, phenotype determination, and sequencing

Isolates were retrieved from existing strain banks in Pakistan Railway General Hospital, Rawalpindi, Pakistan; the Pakistan Institute of Medical Sciences in Islamabad, Pakistan; or Barnes Jewish Hospital/Washington University (WU) School of Medicine in Saint Louis, Missouri, U.S.A. Isolate phenotypes and draft genome sequences were determined in previous work (Pesesky et al., 2015) (BioProject ID PRJNA261540). Briefly, antibiotic susceptibility phenotypes were determined using Kirby-Bauer disk diffusion according to Clinical and Laboratory Standards Institute guidelines and interpretive criteria (CLSI, 2015). Twelve antibiotics were tested: ampicillin (AMP), cefazolin (CFZ), cefotetan (CTT), ceftazidime (CAZ), ceftriaxone (CRO), cefepime (FEP), meropenem (MEM), ciprofloxacin (CIP), trimethoprim-sulfamethoxazole (SXT), gentamicin (GEN), doxycycline (DOX), chloramphenicol (CHL). Each isolate was sequenced on the Illumina Hi Seq 2500 using 101 bp paired-end reads, and reads were assembled into draft genomes using Velvet (Zerbino and Birney, 2008).

### Antibiotic resistance gene identification

Open reading frames in the genome assemblies were identified using GeneMark software (Borodovsky and Lomsadze, 2011) using the command: “gmhmmp -m < model_name> -o <outfile> -a <contig_name_file>.” Genes were separately annotated by three antibiotic resistance databases: ResFinder (Zankari et al., 2012), the Comprehensive Antibiotic Resistance Database (CARD) (McArthur et al., 2013), and Resfams (Gibson et al., 2015). These three databases represent three different options for cataloging resistance gene sequence data: a gene nucleotide sequence database, a gene product amino-acid sequence database, and a gene product amino acid Hidden Markov Model (HMM) database.

All protein sequences from each draft genome were compared to the ResFinder database using BLAST+ command line software downloaded from the National Center for Biotechnology Information (NCBI, https://blast.ncbi.nlm.nih.gov/Blast.cgi) using the command “tblastn -query <Sequence_file> -db <ResFinder_database_name> -culling_limit 1 -outfmt '6 qseqid sseqid length qlen slen qstart qend pident' -out <out_file>.” BLAST+ was also used to compare isolate genomes to the CARD database, using the command “blastp -query <protein_sequence_file> -db <CARD_name> -culling_limit 1 -outfmt '6 qseqid sseqid length qlen slen qstart qend pident' -out <Output_file>.” In both cases the “-culling_limit 1” flag indicates that only the best match for each query protein will be kept. Results were filtered to remove matches over less than 60% of the query sequence length, and to remove duplicate matches within the results for each genome.

Comparisons to the Resfams HMM database were made using HMMER3 (Eddy, 2009) with the command “hmmscan –cut_ga -o /dev/null –tblast <target_out_file> –domtblast <domain_out_file> <database_file> <protein_input_file>.” As in previous analysis of these genomes (Pesesky et al., 2015), genes were also compared with the Pfams (Finn et al., 2014) and TIGRFAMs (Haft et al., 2001) HMM databases and results from all three HMM databases were concatenated into a single file for analysis.

### Antibiotic resistance prediction

Antibiotic susceptibility was predicted for each isolate using a rules-based (RB) algorithm and a Logistic Regression (LR) algorithm with Resfams annotated genes as the inputs. The *in vitro* phenotypic susceptibility results were used as the gold standard for comparison with our GBASP results. Errors in the GBASP were defined in relation to the *in vitro* results, with major errors being a resistant prediction from the GBASP being discrepant with an *in vitro* susceptible phenotype and very major errors being a susceptible prediction from the GBASP being discrepant with an *in vitro* resistant phenotype. For the purposes of this comparison, intermediate resistance phenotypes from the phenotypic AST were counted as resistant. The RB algorithm was built with five separate algorithms, described below, using custom python scripts available on (https://github.com/mpesesky/susceptibility-prediction). For the LR algorithm, inputs for each organism were the species, the resistance genes as determined by resfams, and the phenotypic value determined *in vitro*. We gave these inputs to the Weka 3 (Hall et al., 2009) data mining software package with a ridge log-likelihood of 1 × 10^−8^ and 10 iterations to find the maximum likelihood using the following parameters: weka.classifiers.functions.Logistic -R 1.0E-8 -M 10. The prediction model built by logistic regression was evaluated by leave-one-out cross validation (Molinaro et al., 2005) where each input genome was separately evaluated by a training set built from the other input genomes.

#### β-lactam antibiotics

Resistance against the β-lactam antibiotic class in the *Enterobacteriaceae* is most frequently mediated by acquired β-lactam cleaving enzymes active against some subset of β-lactam antibiotics. We identified each specific β-lactamase in a given isolate, and matched that identity to the set of β-lactam antibiotics to which it was expected to give resistance (http://www.lahey.org/Studies, accessed March 25th, 2014; Bush and Jacoby, 2010). For beta-lactamases that were identified that had less than 100% amino acid identity to a known beta-lactamase, we assigned it to the resistance profile of the known β-lactamase with highest sequence similarity. The expected resistance profile for the isolate was the union of all individual β-lactamase sets, and the predicted susceptibility profile was the inverse of the resistance profile. For *E. coli* strains, the chromosomal *ampC* was identified as the class C β-lactamase with less than 96% identity to known, plasmid-borne variants of *ampC*. The contribution to resistance for those genes was considered to be zero, since their expression is known to be too low to provide clinical levels of resistance. For the LR algorithm we used isolate species and the presence or absence of specific β-lactamase families as input.

#### Ciprofloxacin

Clinical levels of resistance to fluoroquinolones in the *Enterobacteriaceae* is most often mediated by mutations in the target genes *gyrA, gyrB*, and *parC*, though subclinical levels of resistance can be mediated by acquired enzymes, such as *qnr*. We predicted susceptibility to ciprofloxacin by comparison of the quinolone resistance determining regions (QRDR) of *gyrA* (residues 68–106), *parC* (residues 68–106), and *gyrB* (residue 426) for each isolate against the wild type. We also identified *qnr* gyrase protection proteins using Resfams. For the RB algorithm, ciprofloxacin resistance was predicted for an isolate only if it contained two or more QRDR mutations across all three targets, or if it contained one QRDR mutation and a *qnr* gene. If the *gyrA* or *parC* genes were not completely assembled and resistance could not be definitively determined, than the resistance prediction was not determined (N.D.). N.D. resistance was not counted as a major error or very major error, but did reduce overall prediction accuracy. For the LR algorithm, isolate species and variations in the QRDR were used as inputs. Because of the large number of input variables in the ciprofloxacin predictions, we tested the effect of attribute selection on our predictive power. We used the CFS (Correlation based Feature Selection) attribute selection algorithm to avoid the potential for bias that a wrapper algorithm would have with a small dataset. The attribute selection command was weka.filters.supervised.attribute.AttributeSelection -E“weka.attributeSelection.CfsSubsetEval” -S“weka.attributeSelection.GreedyStepwise -T-1.7976931348623157E308 -N -1.”

#### Doxycycline and chloramphenicol

For the RB algorithm we predicted isolates to be susceptible to doxycycline only in the absence of any genes with known tetracycline resistance phenotypes (ex. *tetA*). The same metric was used for chloramphenicol, where examples of chloramphenicol resistance genes include chloramphenicol acetyl-transferases and chloramphenicol efflux pumps. The LR algorithm for both antibiotic conditions used isolate species and resistance gene family identity as inputs.

#### Gentamicin

Like the β-lactams, resistance to aminoglycoside antibiotics in the *Enterobacteriaceae* is primarily mediated by specific enzymes that affect a subset of aminoglycosides. For the RB algorithm we determined susceptibility to gentamicin by comparing the sequence of identified aminoglycoside resistance genes against a database of known profiles adapted from CARD. If any of an isolate's aminoglycoside resistance genes had previously been reported to provide resistance to gentamicin, we predicted the isolate to be resistant; otherwise we predicted it to be susceptible. For the LR algorithm we used isolate species and presence or absence of each resistance gene family identity as inputs.

#### Trimethoprim-sulfamethoxazole

Resistance to the combination therapy trimethoprim-sulfamethoxazole in the *Enterobacteriaceae* is primarily mediated by acquired resistant variants of the dihydrofolate-reductase (DHFR) target enzyme. We are not aware of a comprehensive collection of resistant and susceptible variants of DHFR, so we focused on identifying isolates that had multiple, unique DHFR variants. For the RB algorithm, we predicted susceptibility to trimethoprim-sulfamethoxazole by enumerating the unique DHFR enzymes present within each isolate, defining unique as having less than 95% amino acid identity to any other DHFR in the genome. If two divergent DHFRs were present within the same genome, that isolate was predicted to be resistant, otherwise it was predicted to be susceptible. For the LR algorithm we clustered all of the DHFR genes at 95% amino acid identity using cd-hit (Fu et al., 2012) generating 20 clusters. We used isolate species and the presence or absence of each DHFR cluster as inputs. The *sul* sulfonamide resistance genes were not detected in any isolate.

### Functional metagenomics

For functional metagenomics (Rondon et al., 2000) we utilized protocols detailed previously (Forsberg et al., 2014). Briefly, we sheared genomic DNA from each isolate and separated the fragments by gel electrophoresis, purifying only the fragments between 2 and 5 kb in size. We then barcoded and pooled the genome fragments in to 7 pools to simplify additional processing. The pooled fragments were next ligated them into the expression vector pZE21 using blunt-end ligation, and transformed into a susceptible *E. coli* DH10B derivative MegaX (ThermoFisher catalog number C640003). We then plated these recombinant *E. coli* libraries onto 11 Mueller-Hinton agar plates, each containing one of the following antibiotics: penicillin (128 μg/ml), amoxicillin (16 μg/ml), cefotaxime (8 μg/ml), ceftazidime (16 μg/ml), meropenem (16 μg/ml), aztreonam (8 μg/ml), gentamicin (16 μg/ml), tetracycline (8 μg/ml), tigecycline (2 μg/ml), chloramphenicol (8 μg/ml), and trimethoprim (8 μg/ml). Antibiotic concentrations were determined as the empiric MIC of the host *E. coli*. We next collected the surviving colonies from each plate and isolated their plasmid DNA. These selected plasmids were then sequenced on the Illumina HiSeq 2500 platform to identify the antibiotic resistance gene that permitted survival. Reads from individual genomes were separated by barcode. Rather than using *de novo* assembly of the reads, as in Forsberg et al. (2014), we aligned reads from each selection to the previously sequenced draft genomes of each of the 78 isolates using the default parameters of Bowtie2 (Langmead and Salzberg, 2012). Genes from those genomes that were fully covered by reads from the selection were retrieved and annotated using the Pfams, TIGRFAMs, and Resfams HMM databases, using HMMER3 (Eddy, 2009) with the command “hmmscan –cut_ga -o /dev/null –tblast <target_out_file> –domtblast <domain_out_file> <database_file> <protein_input_file>.” Results from all three HMM databases were concatenated into a single file for analysis.

## Results

### Resistance gene database performance

**Figure 1:**
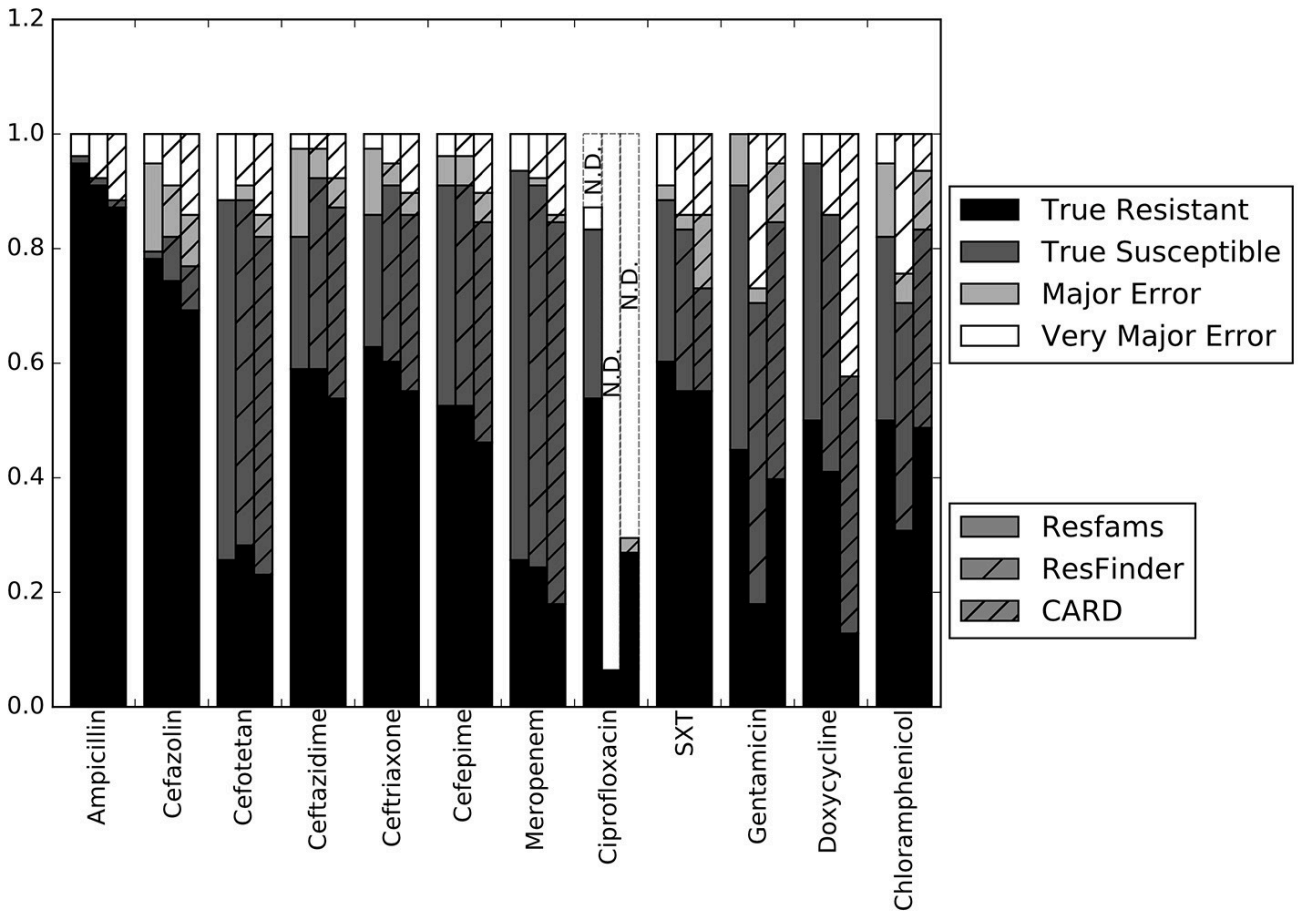
**Prediction accuracy of the RB algorithm using the Resfams, CARD, or ResFinder databases**. True Resistant: both the prediction algorithm and the gold standard AST returned “resistant.” True Susceptible: both the prediction algorithm and gold standard AST returned “susceptible.” Major Error: the prediction algorithm returned “resistant” while the gold standard AST returned “susceptible.” Very Major Error: the prediction algorithm returned “susceptible” while the gold standard AST returned “resistant.” SXT, trimethoprim-sulfamethoxazole. N.D. Susceptibility could not be predicted for this antibiotic in these isolates.

Our first step was to compare the performance of the different resistance gene databases in the context of susceptibility prediction. Since this study used clinical isolates rather than well-characterized lab strains or simulated data, there does not exist a definitive set of resistance genes that should have been detected by comparisons to each database. For this reason, we evaluated each database by running the resistance genes it identified through the RB prediction algorithm, and comparing the GBASP results for each isolate to the phenotypic AST results (Figure 1). Overall, the predictions based on the Resfams database were the closest to the phenotypic results, averaging 89.0% agreement across all 12 antibiotics. Predictions based on ResFinder were the next closest, with 85.4% agreement, and those based on CARD had the least agreement with the phenotypic results at 81.0%.

The largest apparent difference between the three algorithms was in their ability to predict resistance or susceptibility for ciprofloxacin. For many of the isolates, insufficient information was available from either the draft genome sequence or the resistance database to make a resistant or susceptible call, in which case the prediction was “not determined.” This occurred in isolates that otherwise appeared to be susceptible, if the QRDR of the *gyrA, gyrB*, or *parC* gene sequences could not be resolved. “Not determined” results were not included in the overall accuracy values given above. Because the Resfams annotations were integrated with Pfams and TIGRfams, it was much more likely to identify housekeeping gene sequences present in the genome. Even discounting the ciprofloxacin results, the RB algorithm based on Resfams annotations had the highest agreement (88.5%) with the phenotypic AST. Agreement for the RB algorithm based on annotations from ResFinder (85.3%) and CARD (80.8%) also remained similar when ciprofloxacin results were excluded.

The results for each of the three databases have similar accuracy across the β-lactam antibiotics and trimethoprim-sulfamethoxazole (Figure 1). For gentamicin and chloramphenicol, Resfams and CARD both had accuracies greater than 80%, while ResFinder was accurate for only 70.5% of isolates. For doxycycline, Resfams had an accuracy of 94.9%, ResFinder had an accuracy of 85.9%, and CARD had an accuracy of 57.7%. We chose to use the Resfams annotations for all subsequent analyses, because they led to the highest overall agreement between the RB algorithm and phenotypic AST, and because agreement did not drop below 75% for any of the antibiotics tested (Figure 1).

### Resistance characteristics of the isolates

**Figure 2:**
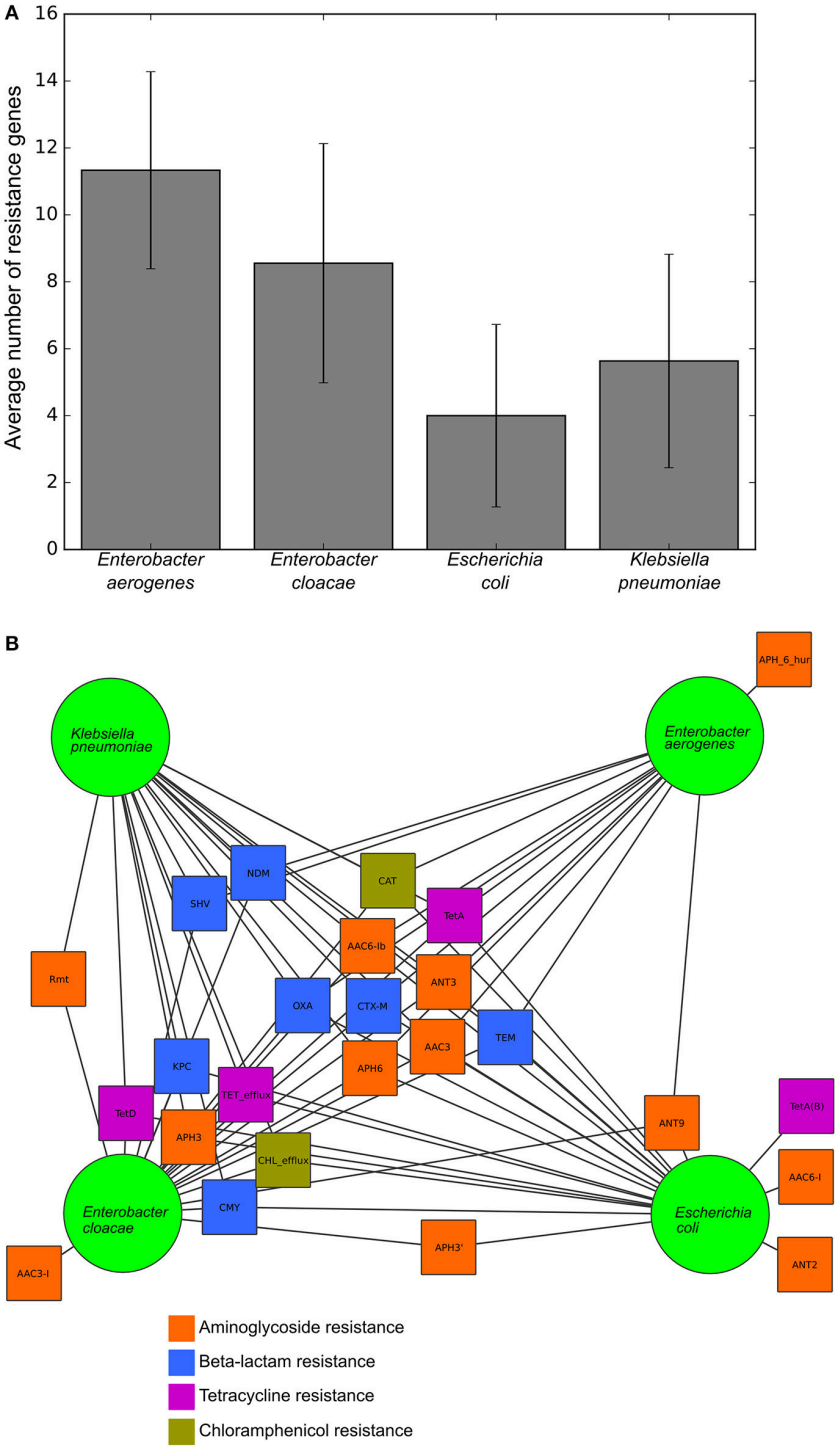
**Resistance gene content and sharing of the tested isolates, (A)** Average number of resistance genes per genome in each of the four species tested. **(B)** Network diagram demonstrating gene sharing between the four species. Each square represents a resistance gene family colored by class of antibiotic. A line between a gene family and a species indicates that the resistance gene family was found within at least one isolate from that species. Gene families were manually clustered based on the species in which they were found.

To validate this isolate set for testing the RB and LR algorithms, we next used Resfams to analyze the specific classes of resistance genes present in each isolate. Many of these isolates were multidrug resistant by the phenotypic AST, and several were resistant to all antibiotics tested (Table 1). Though the average number of resistance genes varied between the species in this set, 9 of the 25 resistance gene families found in these organisms were found in all four species, with only 5 families present in one species exclusively (Figure 2). Previous work with these isolates demonstrated that identical resistance genes within these gene families were shared between different bacterial species (Pesesky et al., 2015), prompting our grouping together of the four species considered into a single test set.

**Table 1:** **Phenotypic resistance profiles**.

**Isolate**	**Species**	**AMP**	**CAZ**	**CTT**	**CFZ**	**CRO**	**FEP**	**MEM**	**CIP**	**SXT**	**GEN**	**DOX**	**CHL**
PH100	*E. coli*	6 (R)	20 (I)	30 (S)	28 (S)	29 (S)	27 (S)	30 (S)	30 (S)	6 (R)	24 (S)	6 (R)	6 (R)
PH101-2	*E. coli*	6 (R)	6 (R)	31 (S)	16 (R)	6 (R)	16 (I)	33 (S)	6 (R)	6 (R)	24 (S)	6 (R)	25 (S)
PH105	*E. coli*	6 (R)	21 (I)	33 (S)	30 (S)	35 (S)	35 (S)	33 (S)	28 (S)	6 (R)	6 (R)	6 (R)	6 (R)
PH108	*E. coli*	6 (R)	6 (R)	6 (R)	6 (R)	6 (R)	17 (I)	28 (S)	6 (R)	19 (S)	24 (S)	6 (R)	17 (I)
PH114	*E. coli*	6 (R)	6 (R)	25 (S)	14 (R)	6 (R)	14 (R)	30 (S)	6 (R)	6 (R)	6 (R)	6 (R)	6 (R)
PH118	*E. coli*	6 (R)	6 (R)	31 (S)	17 (R)	6 (R)	19 (S)	32 (S)	6 (R)	17 (S)	6 (R)	6 (R)	23 (S)
PH129	*E. coli*	6 (R)	6 (R)	26 (S)	14 (R)	6 (R)	13 (R)	29 (S)	6 (R)	6 (R)	6 (R)	10 (R)	23 (S)
PH135	*E. coli*	6 (R)	6 (R)	30 (S)	23 (S)	6 (R)	21 (S)	33 (S)	26 (S)	29 (S)	25 (S)	20 (S)	27 (S)
PH141	*E. coli*	16 (I)	22 (I)	28 (S)	28 (S)	31 (S)	32 (S)	32 (S)	6 (R)	28 (S)	26 (S)	20 (S)	27 (S)
PH143	*E. coli*	6 (R)	6 (R)	6 (R)	6 (R)	6 (R)	6 (R)	28 (S)	6 (R)	6 (R)	6 (R)	6 (R)	25 (S)
PH151-2	*E. coli*	6 (R)	6 (R)	29 (S)	19 (I)	6 (R)	17 (I)	32 (S)	6 (R)	6 (R)	6 (R)	13 (I)	26 (S)
PH156-1	*E. coli*	6 (R)	6 (R)	26 (S)	16 (R)	9 (R)	15 (I)	30 (S)	6 (R)	6 (R)	20 (S)	19 (S)	6 (R)
PH18	*E. coli*	6 (R)	6 (R)	27 (S)	17 (R)	6 (R)	14 (R)	28 (S)	6 (R)	20 (S)	21 (S)	11 (I)	22 (S)
PH20	*E. coli*	6 (R)	6 (R)	6 (R)	6 (R)	6 (R)	6 (R)	27 (S)	6 (R)	6 (R)	6 (R)	11 (I)	6 (R)
PH31	*E. coli*	6 (R)	6 (R)	28 (S)	18 (I)	6 (R)	16 (I)	29 (S)	6 (R)	6 (R)	6 (R)	19 (S)	24 (S)
PH39	*E. coli*	6 (R)	21 (I)	32 (S)	31 (S)	32 (S)	35 (S)	32 (S)	26 (S)	6 (R)	23 (S)	10 (R)	25 (S)
PH51	*E. coli*	6 (R)	15 (R)	28 (S)	26 (S)	28 (S)	30 (S)	29 (S)	6 (R)	25 (S)	27 (S)	6 (R)	19 (S)
PH5	*E. coli*	6 (R)	6 (R)	28 (S)	21 (S)	12 (R)	21 (S)	29 (S)	6 (R)	6 (R)	21 (S)	12 (I)	23 (S)
PH85	*E. coli*	6 (R)	22 (R)	30 (S)	29 (S)	32 (S)	34 (S)	32 (S)	26 (S)	6 (R)	24 (S)	6 (R)	21 (S)
PH90	*E. coli*	6 (R)	6 (R)	29 (S)	23 (S)	6 (R)	24 (S)	31 (S)	6 (R)	6 (R)	22 (S)	6 (R)	25 (S)
PH92-1	*E. coli*	6 (R)	20 (I)	31 (S)	31 (S)	31 (S)	32 (S)	31 (S)	30 (S)	6 (R)	24 (S)	6 (R)	6 (R)
PH93	*E. coli*	6 (R)	20 (R)	31 (S)	29 (S)	30 (S)	32 (S)	30 (S)	27 (S)	6 (R)	24 (S)	6 (R)	6 (R)
PH94	*E. coli*	6 (R)	6 (R)	27 (S)	18 (I)	6 (R)	19 (S)	30 (S)	6 (R)	6 (R)	6 (R)	6 (R)	25 (S)
PH98	*E. coli*	6 (R)	20 (R)	28 (S)	25 (S)	29 (S)	26 (S)	29 (S)	30 (S)	6 (R)	24 (S)	6 (R)	6 (R)
WU31	*E. coli*	6 (R)	6 (R)	9 (R)	10 (R)	8 (R)	12 (R)	12 (R)	6 (R)	6 (R)	21 (S)	9 (R)	6 (R)
WU32	*E. coli*	6 (R)	6 (R)	14 (I)	14 (R)	12 (R)	17 (R)	18 (R)	6 (R)	6 (R)	17 (S)	6 (R)	6 (R)
WU33	*E. coli*	6 (R)	6 (R)	15 (I)	14 (R)	10 (R)	15 (R)	14 (R)	36 (S)	20 (S)	12 (R)	20 (S)	6 (R)
WU34	*E. coli*	6 (R)	6 (R)	12 (R)	6 (R)	6 (R)	6 (R)	15 (R)	6 (R)	6 (R)	6 (R)	6 (R)	14 (I)
WU35	*E. coli*	16 (I)	16 (I)	13 (I)	13 (R)	15 (R)	23 (S)	21 (I)	31 (S)	6 (R)	17 (S)	27 (S)	28 (S)
WU40	*E. coli*	6 (R)	11 (R)	22 (S)	21 (S)	25 (S)	32 (S)	30 (S)	34 (S)	25 (S)	21 (S)	20 (S)	24 (S)
WU43	*E. coli*	19 (S)	24 (S)	30 (S)	30 (S)	28 (S)	34 (S)	33 (S)	36 (S)	28 (S)	22 (S)	22 (S)	27 (S)
WU44	*E. coli*	6 (R)	12 (R)	21 (S)	22 (S)	26 (S)	31 (S)	30 (S)	6 (R)	25 (S)	21 (S)	23 (S)	26 (S)
WU45	*E. coli*	6 (R)	21 (I)	29 (S)	28 (S)	29 (S)	33 (S)	30 (S)	6 (R)	19 (S)	21 (S)	22 (S)	27 (S)
PH112-2	*E. coli*	6 (R)	6 (R)	13 (I)	6 (R)	6 (R)	14 (R)	14 (R)	14 (R)	6 (R)	24 (S)	9 (R)	6 (R)
PH113	*E. aero*	6 (R)	6 (R)	14 (I)	6 (R)	6 (R)	14 (R)	13 (R)	14 (R)	6 (R)	23 (S)	9 (R)	6 (R)
PH134	*E. aero*	6 (R)	6 (R)	13 (I)	6 (R)	6 (R)	12 (R)	13 (R)	15 (R)	6 (R)	6 (R)	12 (I)	6 (R)
PH138-2	*E. aero*	6 (R)	6 (R)	13 (I)	6 (R)	6 (R)	12 (R)	13 (R)	14 (R)	6 (R)	6 (R)	9 (R)	6 (R)
PH63	*E. aero*	6 (R)	6 (R)	13 (I)	6 (R)	6 (R)	12 (R)	14 (R)	14 (R)	6 (R)	6 (R)	6 (R)	6 (R)
PH84-2	*E. aero*	6 (R)	6 (R)	12 (R)	6 (R)	6 (R)	12 (R)	13 (R)	14 (R)	6 (R)	6 (R)	9 (R)	6 (R)
PH112-1	*E. aero*	6 (R)	6 (R)	26 (S)	16 (R)	6 (R)	16 (I)	29 (S)	16 (I)	6 (R)	6 (R)	16 (S)	6 (R)
PH125	*E. cloa*	6 (R)	6 (R)	6 (R)	6 (R)	6 (R)	9 (R)	13 (R)	6 (R)	23 (S)	6 (R)	15 (S)	9 (R)
PH158	*E. cloa*	6 (R)	6 (R)	6 (R)	6 (R)	6 (R)	12 (R)	14 (R)	6 (R)	6 (R)	6 (R)	10 (R)	6 (R)
PH23	*E. cloa*	6 (R)	6 (R)	6 (R)	6 (R)	6 (R)	10 (R)	16 (R)	6 (R)	6 (R)	6 (R)	10 (R)	6 (R)
PH24-2	*E. cloa*	6 (R)	6 (R)	6 (R)	6 (R)	6 (R)	9 (R)	15 (R)	6 (R)	6 (R)	6 (R)	11 (I)	6 (R)
PH82	*E. cloa*	6 (R)	6 (R)	22 (S)	6 (R)	6 (R)	9 (R)	29 (S)	6 (R)	6 (R)	6 (R)	10 (R)	22 (S)
WU26	*E. cloa*	6 (R)	6 (R)	6 (R)	6 (R)	9 (R)	13 (R)	14 (R)	6 (R)	6 (R)	6 (R)	11 (I)	17 (I)
WU27	*E. cloa*	6 (R)	6 (R)	13 (R)	10 (R)	12 (R)	14 (R)	14 (R)	6 (R)	6 (R)	13 (I)	6 (R)	10 (R)
WU29	*E. cloa*	6 (R)	6 (R)	6 (R)	6 (R)	13 (R)	10 (R)	11 (R)	29 (S)	24 (S)	21 (S)	15 (S)	21 (S)
PH102	*E. cloa*	6 (R)	6 (R)	10 (R)	6 (R)	6 (R)	15 (I)	27 (S)	18 (I)	6 (R)	6 (R)	6 (R)	6 (R)
PH10	*K. pneu*	6 (R)	6 (R)	23 (S)	6 (R)	6 (R)	11 (S)	26 (S)	6 (R)	6 (R)	6 (R)	14 (S)	11 (R)
PH11	*K. pneu*	6 (R)	6 (R)	6 (R)	6 (R)	6 (R)	9 (R)	12 (R)	6 (R)	6 (R)	11 (R)	14 (S)	12 (R)
PH124	*K. pneu*	6 (R)	6 (R)	27 (S)	15 (R)	6 (R)	17 (I)	27 (S)	10 (R)	6 (R)	25 (S)	10 (R)	16 (I)
PH12	*K. pneu*	6 (R)	6 (R)	9 (R)	6 (R)	6 (R)	9 (R)	12 (R)	6 (R)	6 (R)	6 (R)	14 (S)	11 (R)
PH139	*K. pneu*	6 (R)	6 (R)	26 (S)	6 (R)	6 (R)	15 (I)	28 (S)	6 (R)	6 (R)	6 (R)	13 (I)	6 (R)
PH150-2	*K. pneu*	6 (R)	6 (R)	23 (S)	6 (R)	6 (R)	10 (R)	27 (S)	6 (R)	6 (R)	24 (S)	6 (R)	6 (R)
PH152	*K. pneu*	6 (R)	6 (R)	24 (S)	6 (R)	6 (R)	8 (R)	26 (S)	6 (R)	6 (R)	22 (S)	6 (R)	6 (R)
PH24-1	*K. pneu*	6 (R)	21 (I)	28 (S)	26 (S)	28 (S)	28 (S)	29 (S)	6 (R)	6 (R)	6 (R)	14 (S)	12 (R)
PH25	*K. pneu*	6 (R)	6 (R)	24 (S)	6 (R)	6 (R)	12 (R)	28 (S)	6 (R)	6 (R)	6 (R)	15 (S)	14 (I)
PH28-1	*K. pneu*	6 (R)	23 (S)	29 (S)	27 (S)	28 (S)	31 (S)	29 (S)	30 (S)	20 (S)	22 (S)	12 (I)	20 (S)
PH38-1	*K. pneu*	10 (R)	23 (S)	29 (S)	26 (S)	28 (S)	31 (S)	29 (S)	30 (S)	20 (S)	23 (S)	12 (I)	19 (S)
PH40	*K. pneu*	9 (R)	23 (S)	30 (S)	27 (S)	29 (S)	32 (S)	30 (S)	30 (S)	21 (S)	24 (S)	13 (I)	18 (S)
PH44	*K. pneu*	6 (R)	6 (R)	6 (R)	6 (R)	6 (R)	9 (R)	12 (R)	6 (R)	11 (I)	6 (R)	15 (S)	23 (S)
PH49-2	*K. pneu*	8 (R)	24 (S)	30 (S)	28 (S)	30 (S)	32 (S)	30 (S)	29 (S)	21 (S)	23 (S)	14 (S)	18 (S)
PH72	*K. pneu*	6 (R)	6 (R)	17 (S)	9 (R)	6 (R)	14 (R)	25 (S)	17 (I)	6 (R)	6 (R)	9 (R)	6 (R)
PH73	*K. pneu*	6 (R)	24 (S)	30 (S)	28 (S)	30 (S)	31 (S)	27 (S)	29 (S)	25 (R)	22 (S)	17 (S)	25 (S)
PH88	*K. pneu*	6 (R)	6 (R)	24 (S)	6 (R)	6 (R)	6 (R)	26 (S)	6 (R)	20 (S)	6 (R)	10 (R)	6 (R)
PH9	*K. pneu*	6 (R)	18 (R)	26 (S)	23 (S)	25 (S)	24 (S)	26 (S)	6 (R)	6 (R)	6 (R)	14 (S)	11 (R)
WU10	*K. pneu*	6 (R)	25 (S)	31 (S)	30 (S)	32 (S)	35 (S)	32 (S)	32 (S)	26 (S)	24 (S)	18 (S)	24 (S)
WU12	*K. pneu*	6 (R)	6 (R)	15 (I)	6 (R)	12 (R)	16 (R)	14 (R)	6 (R)	6 (R)	22 (S)	14 (S)	6 (R)
WU18	*K. pneu*	6 (R)	6 (R)	15 (I)	6 (R)	11 (R)	15 (R)	14 (R)	6 (R)	6 (R)	20 (S)	14 (S)	6 (R)
WU21	*K. pneu*	6 (R)	6 (R)	14 (I)	6 (R)	6 (R)	6 (R)	17 (R)	6 (R)	18 (S)	22 (S)	6 (R)	19 (S)
WU23	*K. pneu*	6 (R)	6 (R)	13 (I)	6 (R)	6 (R)	6 (R)	13 (R)	23 (S)	6 (R)	6 (R)	10 (R)	10 (R)
WU2	*K. pneu*	11 (R)	25 (S)	31 (S)	30 (S)	34 (S)	33 (S)	31 (S)	36 (S)	19 (S)	21 (S)	18 (S)	24 (S)
WU3	*K. pneu*	6 (R)	24 (S)	30 (S)	29 (S)	32 (S)	33 (S)	30 (S)	32 (S)	24 (S)	22 (S)	19 (S)	23 (S)
WU6	*K. pneu*	7 (R)	22 (S)	31 (S)	29 (S)	31 (S)	34 (S)	32 (S)	33 (S)	27 (S)	24 (S)	18 (S)	23 (S)
WU7	*K. pneu*	6 (R)	23 (S)	29 (S)	27 (S)	30 (S)	32 (S)	30 (S)	24 (S)	12 (I)	22 (S)	19 (S)	24 (S)
WU8	*K. pneu*	7 (R)	23 (S)	30 (S)	29 (S)	32 (S)	34 (S)	33 (S)	24 (S)	10 (R)	26 (S)	11 (I)	6 (R)
WU9	*K. pneu*	10 (R)	24 (S)	30 (S)	30 (S)	32 (S)	34 (S)	30 (S)	31 (S)	23 (S)	23 (S)	19 (S)	26 (S)

*Values given as zone of inhibition in mm (interpretation: R = “resistant,” S = “susceptible,” I = “intermediate”). E. aero, Enterobacter aerogenes; E. cloa, Enterobacter cloacae; K. pneu, Klebsiella pneumoniae*.

**Figure 3:**
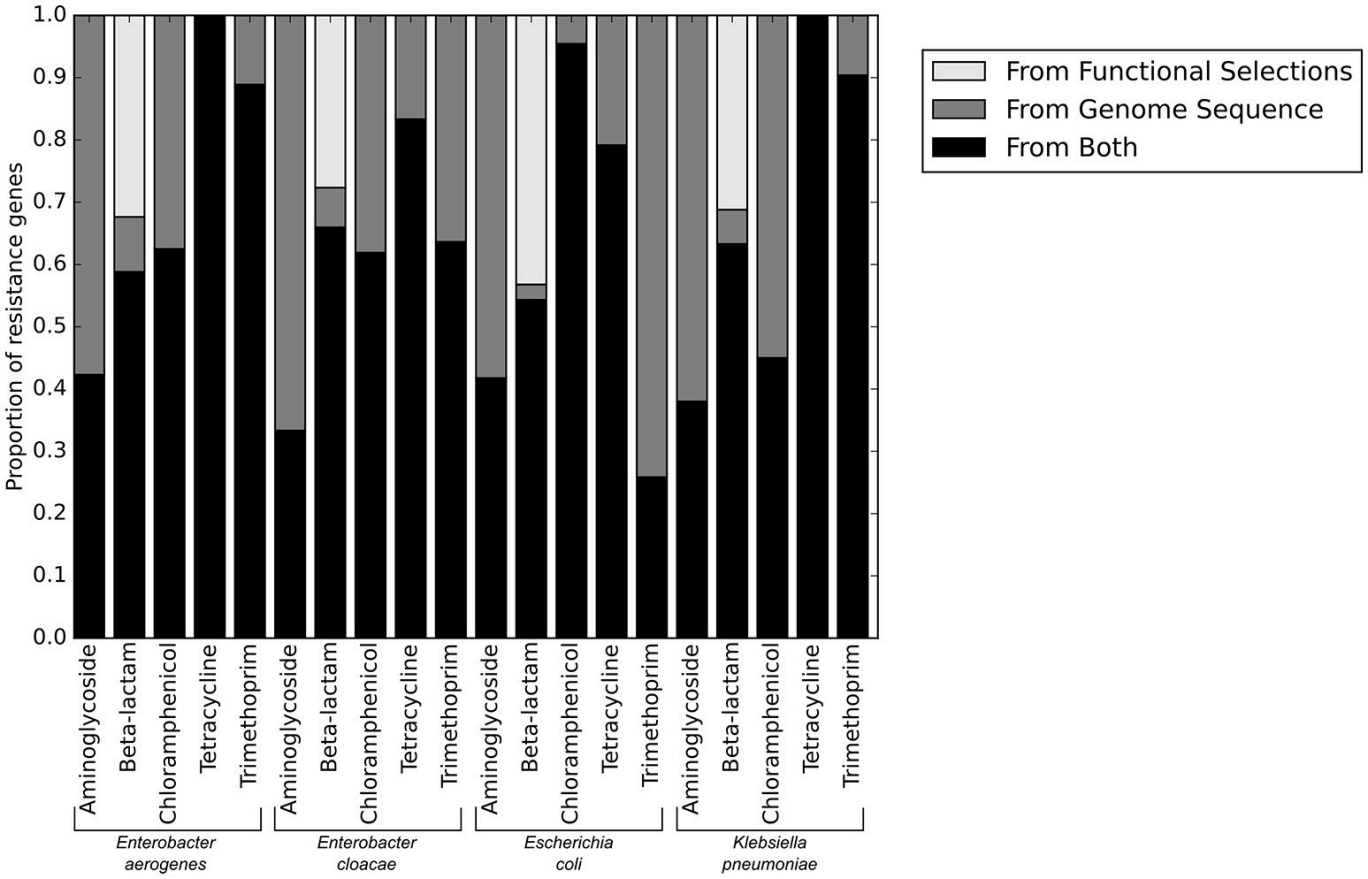
**Resistance genes identified from sequence alone, and those verified functional by functional metagenomics**.

We next used functional metagenomic selections to experimentally test whether our annotations had correctly identified the complete set of resistance genes present in the isolate genomes. Functional metagenomics allows multiple genomes to be simultaneously assayed for genes that provide a specific function. We created shotgun expression libraries in *E. coli* from genomic DNA fragments of the 78 pathogenic isolate genomes, and functionally tested these for antibiotic resistance activities. We sequenced the selected resistance-conferring genomic DNA fragments conferring resistance using short read DNA sequencing on the Illumina platform and aligned the reads to each of the 78 genomes individually (Figure 3).

No resistance genes to ciprofloxacin were identified by functional metagenomics, since the native quinolone targets in the expression strain provide dominant susceptibility even in the context of exogenous expression of alternative resistant targets (Naeem et al., 2016), and the qnr genes present in these genomes were insufficient to provide resistance on their own. Consistent with using gentamicin as our only aminoglycoside resistance selection, only aminoglycoside modifying enzymes specific to gentamicin were identified by functional metagenomics, though aminoglycoside modifying enzymes with other specificities were annotated in the genomes. The only genes providing resistance to trimethoprim-sulfamethoxazole were target dihydrofolate reductases. We anticipated that any DHFR could provide resistance when heterologously overexpressed in a functional metagenomic selection. We were therefore surprised to note that a much lower proportion of *E. coli* DHFR genes were identified in the functional selections than DHFR genes from the other three pathogenic species tested.

The only antibiotic class for which we identified putative resistance genes by functional metagenomics that were not identified from the genome sequence were the beta-lactams. The majority of genes in this category were *ramA* transcriptional regulators which are known to confer resistance when overexpressed in *E. coli* (George et al., 1995), but which are a part of the baseline susceptibility levels of their natural *K. pneumoniae* and *Enterobacter* sp. hosts. Similarly, the chromosomal *ampC* in *E. coli* genome sequences were not included as part of the final phenotypic prediction because they are known to be natively repressed, but when overexpressed (as in functional metagenomics) they provide broad-spectrum resistance. However, variants of *ampC* known to be vector borne and highly expressed (such as the CMY family) were counted as resistance genes when seen in the genome sequence, though they were phenotypically indistinguishable from chromosomal *ampC* genes in the functional metagenomic assay. Accordingly, the functional metagenomic results supported the whole genome resistance gene identification, and did not identify any novel genes that could have caused errors in the predictions.

### Comparing the RB and LR algorithms

**Figure 4:**
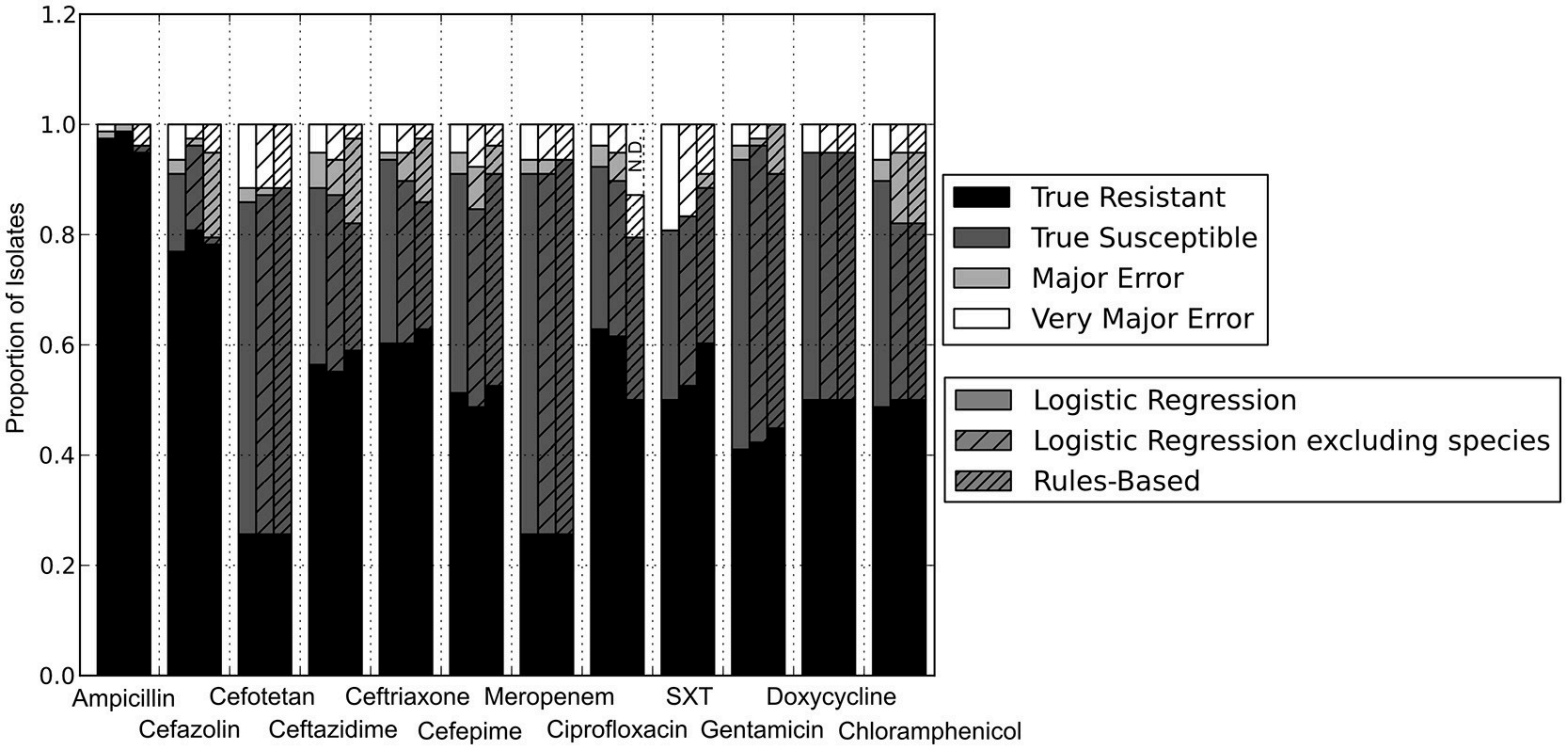
**Prediction accuracy of GBASP algorithms**. True Resistant: both the prediction algorithm and the gold standard AST returned “resistant.” True Susceptible: both the prediction algorithm and gold standard AST returned “susceptible.” Major Error: the prediction algorithm returned “resistant” while the gold standard AST returned “susceptible.” Very Major Error: the prediction algorithm returned “susceptible” while the gold standard AST returned “resistant.” SXT, trimethoprim-sulfamethoxazole. N.D. Susceptibility could not be predicted for this antibiotic in these isolates.

**Figure 5:**
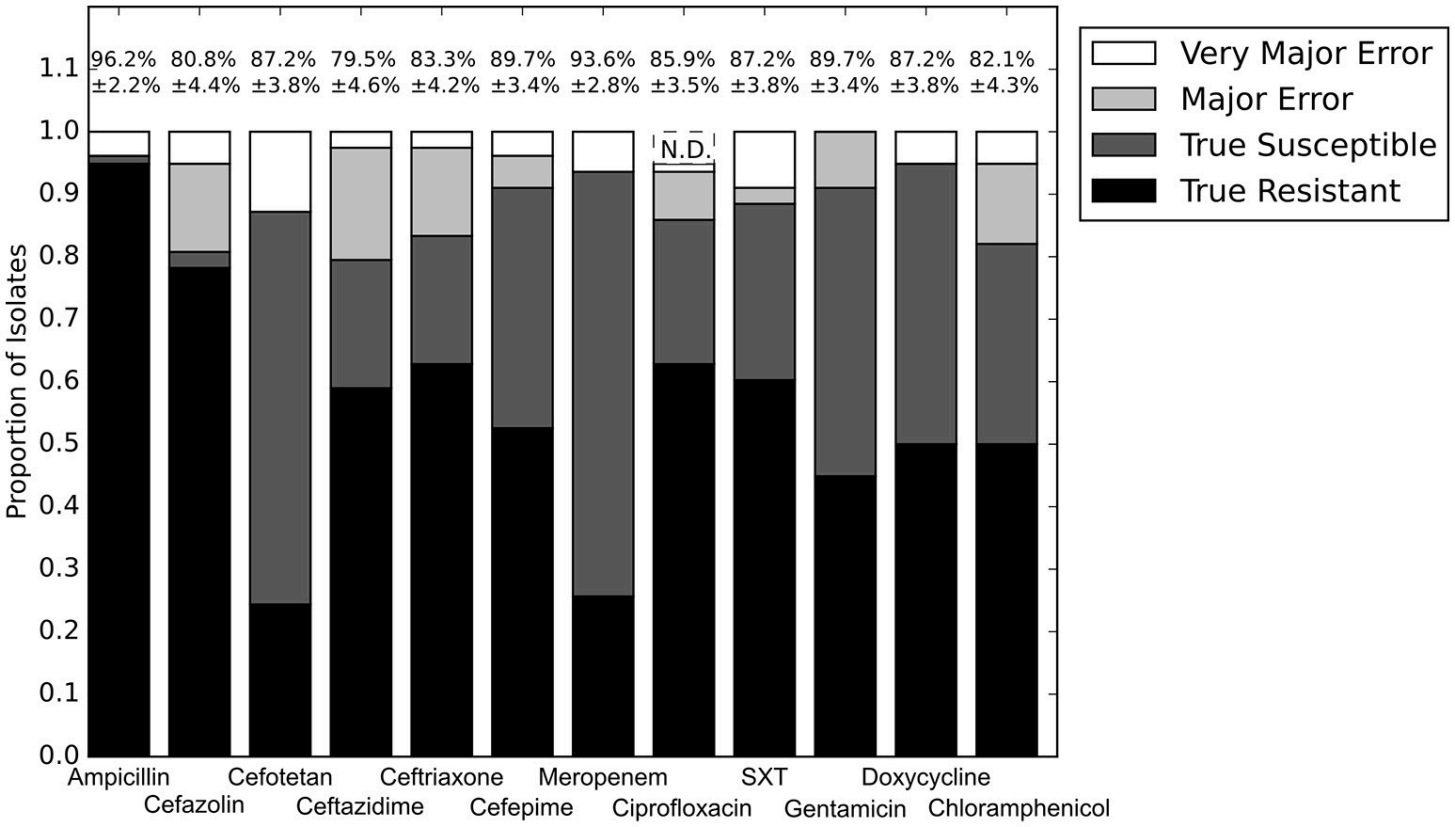
**Prediction accuracy for RB algorithm alone**. Percentages above bars represent percent accurate predictions and standard error for accuracy percentage. SXT, trimethoprim-sulfamethoxazole. N.D. Susceptibility could not be predicted for this antibiotic and these isolates.

**Figure 6:**
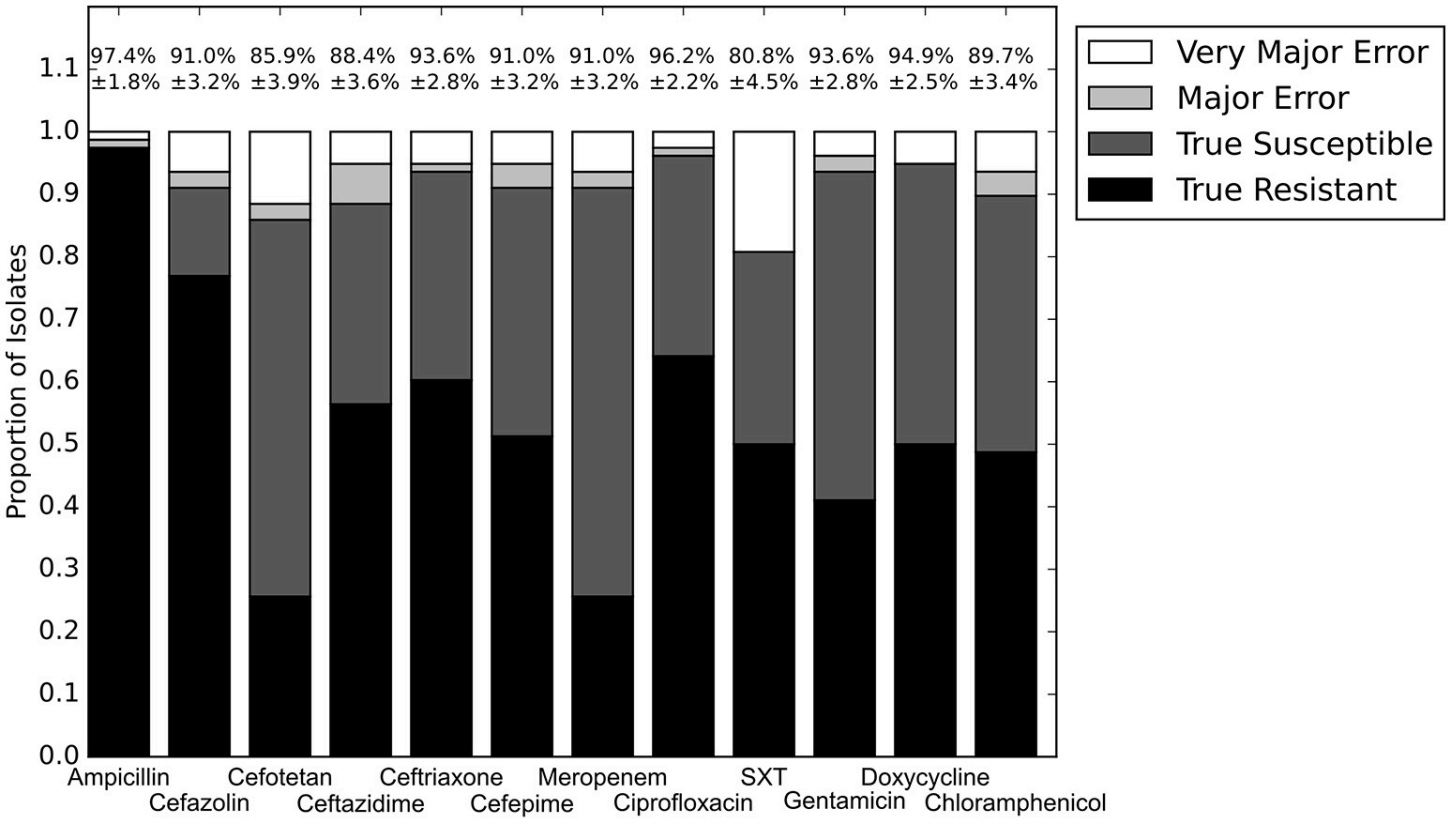
**Prediction for LR algorithm alone**. Percentages above bars represent percent accurate predictions and standard error for accuracy percentage. SXT, trimethoprim-sulfamethoxazole.

**Figure 7:**
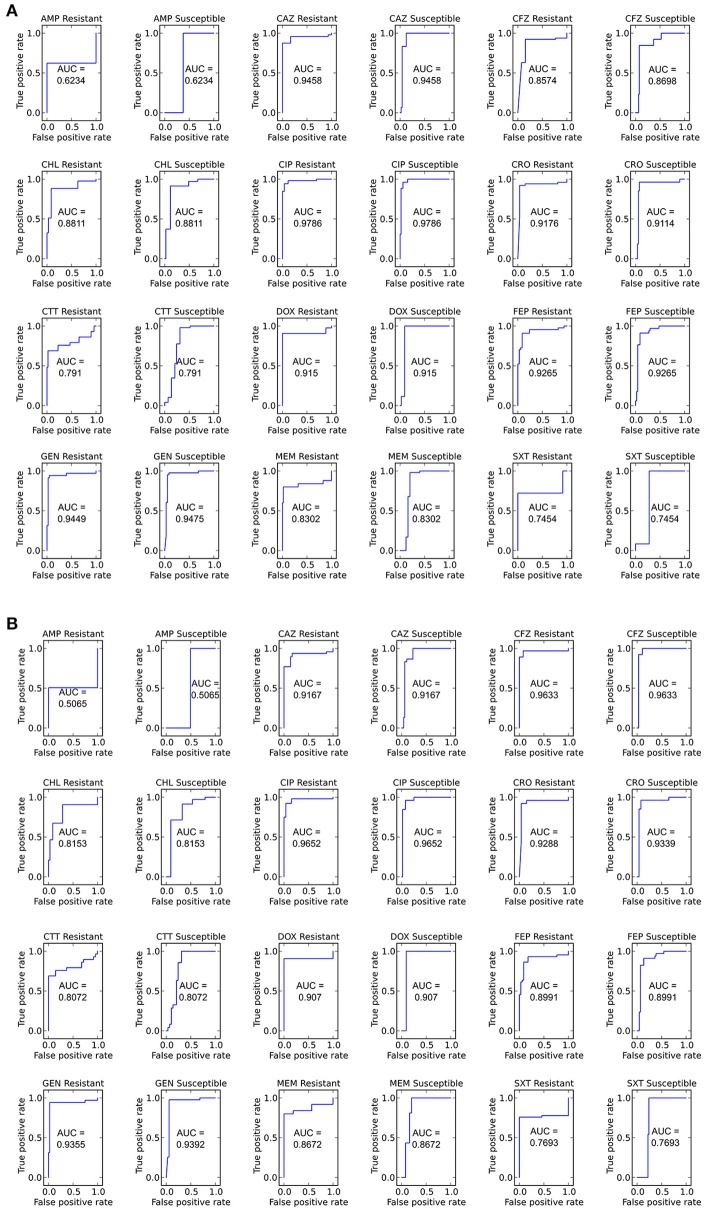
**ROC curves for predicting susceptible and resistant isolates for each antibiotic using the LR algorithm (A)** Including and **(B)** not including species as an input. Area under the curve (AUC) is given for each ROC curve.

With the set of resistance genes confirmed, we applied LR machine learning and RB algorithms to predict antibiotic susceptibility for each isolate, using their *in vitro* measured phenotypes as the gold standard (Figure 4). Our RB algorithm had overall agreement of 89.0% with the phenotypic AST, with an overall major error rate of 6.0% and very major error rate of 4.9%. Agreement for individual antibiotics ranged from 79.5 to 96.2% while major error rates ranged from 0 to 15.4% and very major error rates ranged from 0 to 11.5% (Figure 5). The LR algorithm had a higher overall agreement of 90.8% to the phenotypic ASTs, with a major error rate of 2.6% and a very major error rate of 6.6% (Figure 4). Individual antibiotic agreement ranged from 80.8 to 97.4%, while major error rates ranged from 0 to 6.4% and very major error rates ranged from 1.3 to 19.2% (Figure 6). The intra-class correlation coefficient for repeat analysis of this data set was 1 for both algorithms, indicating perfect reproducibility. Receiver operating characteristic (ROC) curves for the prediction models produced by the LR algorithm showed area under the curve values over 0.9 for half of the antibiotics tested (Figure 7A). Though the rules-based approach was biased to produce major errors rather than very major errors in the case of ambiguity, the logistic regression algorithm was not biased toward either error type. Despite this, the very major error rates were similar between the two algorithms [RB: 4.8%, 95% confidence interval (CI) 2.8 to 6.7%; LR: 6.6%, 95% CI 4.0 to 9.2%]. Both algorithms correctly identified susceptible and resistant organisms for each of the 12 antibiotics tested.

**Figure 8:**
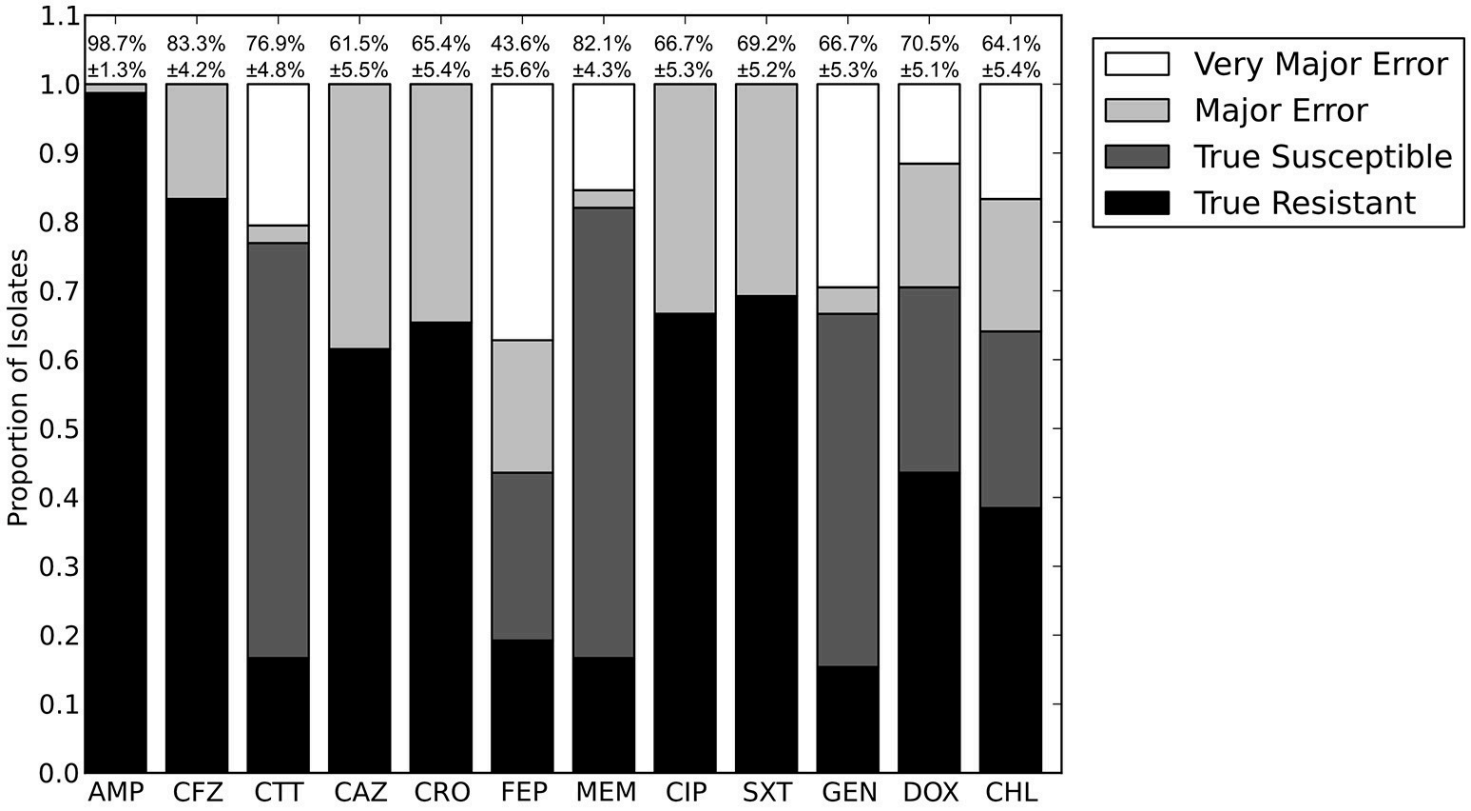
**Prediction accuracy for LR algorithm using species as the only input variable**. AMP, ampicillin; CFZ, cefazolin; CTT, cefotetan; CAZ, ceftazidime; CRO, ceftriaxone; FEP, cefepime; MEM, meropenem; CIP, ciprofloxacin; SXT, trimethoprim-sulfamethoxazole; GEN, gentamicin; DOX, doxycycline; CHL, chloramphenicol.

We next sought to determine if species-specific factors contributed predictive power to the LR algorithm, or if most of the predictive power came from species-independent variables, such as the presence of resistance genes or mutations in conserved enzymes, identified across species. To make this distinction we repeated the LR predictions either excluding species as a variable (Figure 4) or using species as the only variable (Figure 8). While species information appeared to have some predictive value as a variable on its own (70.7% accurate predictions, with 18.4% major errors and 10.9% very major errors), it was not independent from the predictive value of the factors shared between species, as the accuracy percentage of the complete model dropped only a small amount when the species variable was excluded (90.1% with a major error rate of 3.7% and a very major error rate of 6.2%, Figure 7B).

### Attribute selection for ciprofloxacin

The RB algorithm for predicting ciprofloxacin resistance was based on the quinolone resistance-determining region (QRDR) of the *gyrA* and *gyrB* DNA gyrase and *parC* topoisomerase genes, as well as presence of the *qnr* quinolone resistance gene. The *oqxAB* quinolone efflux pump genes were also detected in *Klebsiella* and *Enterobacter* genomes, but were not used as inputs in the RB or LR algorithms because they have not been found to provide high levels of resistance (Hawkey and Jones, 2009) and reduced predictive power in this set. The large number of mutations detected in the QRDR of the total set of isolates led to 97 input variables for the LR algorithm for ciprofloxacin. To determine which of these variables most affected the phenotype and to reduce the chance that the large number of variables artificially inflated prediction accuracy for ciprofloxacin, we performed attribute selection. Four variables were shown to be most predictive by attribute selection: the presence or absence of *qnr*, two mutations at serine 83 of GyrA (to threonine and phenylalanine), and one mutation in serine 80 of ParC (to isoleucine). Predictions made using only these four input variables showed a slightly different error profile to predictions made from the full set of 97 input variables, but had the same overall accuracy (92.3%).

### Antibiotic specific sources of error

Doxycycline was unique among the antibiotics tested in that all of the errors for each prediction algorithm were very major errors (Figure 4). Since no novel tetracycline resistance genes were detected in the functional metagenomic experiment (Figure 3), we performed repeat phenotypic AST on the erroneous isolates with three independent testers (Table 2). All but two of these isolates were re-classified as susceptible by all three testers, one was classified as susceptible by two of the three testers, and the last retained its resistant classification for all three testers. While we cannot determine the impact that repeat testing would have had if performed on the full matrix of isolates and antibiotics, in this instance if we had used the repeat values rather than the original values as our gold standard we would have achieved an accuracy of 98.7% for doxycycline.

**Table 2:** **Doxycycline re-test phenotypic profiles**.

	**Initial ZOI**	**Initial Interpretation**	**ZOI A**	**Interpretation A**
WU8	11	Intermediate	10	Resistant
WU26	11	Intermediate	15	Susceptible
PH28-1	12	Intermediate	16	Susceptible
PH38-1	12	Intermediate	16	Susceptible
PH40	13	Intermediate	16	Susceptible
PH139	13	Intermediate	14	Susceptible
	**ZOI B**	**Interpretation B**	**ZOI C**	**Interpretation C**
WU8	11	Intermediate	11	Intermediate
WU26	15	Susceptible	15	Susceptible
PH28-1	17	Susceptible	17	Susceptible
PH38-1	17	Susceptible	17	Susceptible
PH40	17	Susceptible	17	Susceptible
PH139	14	Susceptible	13	Intermediate

Mutation or loss of particular porins, in combination with an extended spectrum beta-lactamase, can enable resistance to fourth generation cephalosporins or even carbapenems. This led to most of the very major errors in our beta-lactam predictions. We could identify these porin deletions by comparing detected porin genes between isolates previously determined to be closely related (Pesesky et al., 2015), but we could not identify resistance causing porin deletions without using that phylogenetic information. Cefotetan represented a special case as only four of the ten very major errors in predicting susceptibility were caused by porin deletions, while the cause of its remaining very major errors could not be determined.

## Discussion

The pathogens used in this study were originally selected to represent a range of antibiotic resistance phenotypes, from fully susceptible to pan resistant (Pesesky et al., 2015). They also represent a high degree of intra-species genetic diversity, particularly for the strains of *E. coli* (Pesesky et al., 2015), though we found that they share many antibiotic resistance families between species as well as between strains. Previous work with these strains identified plasmid sequences from several of the isolates, and we postulate that plasmids were the primary driver of resistance gene sharing between these four species. There was little variation between species in the performance of the GBASP algorithms, likely as a result of this highly shared resistome.

Our set is weighted toward highly-resistant isolates compared to an expected random set of clinical isolates, as highly resistant isolates would be the ones where it is most important to rapidly determine susceptibility phenotypes. Carbapenem resistant *Enterobacteriaceae*, which represented 24 of our 78 isolates, have been designated one of the most urgent antibiotic resistant threats in the U.S. today (CDC, 2013), and so our set represents some of the most challenging pathogens clinicians are faced with from a treatment perspective. The high degree of accuracy of our predictions with this set shows that GBASP will remain a viable option for rapid diagnostics even as the prevalence of antibiotic resistance continues to increase. At the same time, predictions models built by the LR algorithm on this set could potentially overestimate resistance in average clinical isolates, and they should be retested on isolates representing a wider range of antibiotic susceptibilities. Along with its initiative to guide production of WGS antimicrobial resistance diagnostics, the FDA will be providing representative isolate sets to be used for diagnostic development (Sichtig, 2016).

There are several arguments against currently using GBASP methods for initial treatment decisions. There are many unknown parameters affecting the connection between genotype and phenotype in bacteria, and so we cannot universally ascribe functionality to the presence of a gene. At the same time some variation in antibiotic susceptibility is due to factors other than resistance gene presence or absence, such as metabolic activity (Kaldalu et al., 2016). Additionally, GBASP methods, unlike phenotypic methods, provide only a binary “resistant” or “susceptible” determination, limiting the information available to clinicians making difficult treatment decisions. PCR or microarray methods are limited by the design of the assay and detect only a select, pre-determined suite of resistance determinants. Genomic methods have the additional barriers of cost and turnaround time. In the analysis herein, the error rates we identified for this isolate set, containing both highly susceptible and highly resistant organisms, are too high to be acceptable as a primary diagnostic informing treatment. Despite all of these drawbacks the speed of genotypic diagnostics in providing clinically relevant information will likely continue to motivate interest and improvements from both the academic and commercial sectors in GBASP techniques.

To maximize the benefits of GBASP for patients and for antibiotic stewardship efforts, methods for improving susceptibility prediction must be built into technique development. The first question for WGS GBASP will be which resistance database to use. For our analysis we evaluated three curated databases already verified in the literature: a nucleotide sequence database (ResFinder), an amino acid sequence database (CARD), and an amino acid HMM database (Resfams). One possible systematic reason for the relative success of Resfams is that it is inherently hierarchical. This means that when a resistance gene variant could not be conclusively identified, its gene family was identified, rather than the next closest variant. For families where different variants can have different spectrums of resistance, such as the TEM β-lactamases, a family-level identification led the RB-algorithm (and likely the LR-algorithm as well) to make a more conservative prediction. Finally, for most antibiotics tested the difference in results between any two databases were fairly small, and it is possible that the differences in performance were not systematic, and were due to variations particular to this set of isolates.

Once particular genes have been identified, the next decision point, for WGS, PCR, or microarray GBASP, is how to predict overall organism resistance and susceptibility. We tested the two most likely approaches: an algorithm based on current curated scientific knowledge, our RB algorithm, which would mimic how a human expert would approach interpreting the results, or an approach that uses machine learning, our LR algorithm. Both methods performed similarly in this study, but there are three major reasons to believe that machine learning will yield a more viable long-term approach.

First, the *Enterobacteriaceae* represent a best-case pathogen family for use of the RB algorithm, since it is the bacterial family about which antibiotic resistance has been best characterized at a molecular and genomic level. GBASP would be useful for many other pathogens with very different resistance patterns, particularly *Mycobacterium tuberculosis* infections, as has been suggested previously (Koser et al., 2013; Ajbani et al., 2015). The LR algorithm, on the other hand, should perform equally well on any bacterial pathogen for which have sufficient numbers of characterized, banked isolates for training.

Second, as our knowledge of resistance increases or as new resistance genes enter the pathogenic population, the RB algorithm will need to have rules amended or added, and it will become increasingly complex. In contrast, updating the LR algorithm will only require adding new genes to the input list, and the minor increases in complexity caused by additional inputs will be balanced by the training set becoming progressively enlarged with every new isolate tested. In theory, once the training set becomes large enough, whole isolate genomes could be given as inputs, potentially uncovering genomic causes of resistance not yet discovered by conventional means. For instance, variations in regulatory genes that affect antibiotic target or resistance genes could have a moderate affect on phenotype that has not yet been elucidated. If the training set were large enough that all open reading frames could be included as inputs in the machine learning algorithm, then such associations could be identified from the resistance patterns. In our hands, neither approach was computationally intensive, although our study focused on one pre-selected pathogen family, and computational complexity will likely become an issue if susceptibility prediction for GBASP becomes more generally used for more diverse pathogens.

Third, machine learning algorithms can be applied to predict quantitative measures of resistance, such as minimum inhibitory concentrations or zones of inhibition, rather than the binary resistant or susceptible interpretations currently available to genotypic diagnostics. These quantitative measures of resistance provide a real benefit when choosing a treatment for a highly resistant infection, and they should be integrated into GBASP so that information can be delivered as quickly as possible. Our set of isolates was not large enough to attempt such quantitative predictions, but it is clear from the phenotypic range of the isolates (Table 1) that quantitative predictions would reveal a large amount of currently inaccessible information.

Clinical use of GBASP will need to be supported by ongoing evaluation of pathogens for the emergence of novel resistance genes. Functional metagenomics is a useful technique to identify the presence of resistance genes as it can be used to assay a large number of isolates in a single experiment (Sommer et al., 2009; Forsberg et al., 2012). Our functional metagenomic experiments in an *E. coli* host show that the resistance genes identified can be transferred successfully from other *Enterobacteriaceae* species, supporting the high proportion of resistance gene families shared between the species and the minimal effect of species seen in the LR model. This experiment additionally demonstrated that the majority of functional resistance genes in these isolates were also identified from the genome sequence. One of the advantages of functional metagenomics is that it can be used to identify the spectrum of resistance within an antibiotic class. For instance, in the aminoglycosides, only genes that provide resistance to gentamicin were identified by functional metagenomics, though the full set of aminoglycoside resistance genes could be seen in the genome sequences. By performing selections on multiple antibiotics in the same class, researchers can use functional metagenomics to define the spectrum of a novel resistance gene as it is identified (Forsberg et al., 2015).

The direct comparison of GBASP to phenotypic AST in this study revealed several remaining challenges in GBASP development. The largest biological challenge is differential expression for the same gene in different contexts. This is known to be important for the spectrum and resistance level of beta-lactamases (Turton et al., 2006; Castanheira et al., 2014), and it likely affected all of our predictions of resistance. These expression level differences can arise either as a result of gene location (plasmid vs. chromosomal) or regulatory sequences. Based on the high degree of sharing between species, we anticipate that many of the resistance genes identified in this study were plasmid-based. Because they are mobile, plasmid-borne genes are less likely to be part of intricate regulatory networks, and the simple assumption that they are expressed in sufficient quantities to provide resistance is more likely to be accurate. On the other hand, some of the plasmids present in these strains may be present in multiple copies, which could also lead to variations in effectiveness in the same resistance gene in different plasmids from different strains. Promoter strength and regulatory sequences were not accounted for in our algorithms because our set was too small to include those additional variables in the LR algorithm, and the necessary rules for the RB algorithm are not well defined. Further studies will be necessary to determine the extent to which expression level can be accounted for in GBASP. The fastest way to improve our predictive power, especially for machine learning approaches, will be to generate more genomic data with matched phenotypic AST data.

A second, related challenge is in improving our knowledge of the rare resistance factors that may be present in pathogens. We observed errors in beta-lactam resistance prediction due to the fact that changes in outer membrane porins were not included in either the RB or LR algorithms. Similarly, there is a great diversity in chromosomal β-lactamase genes across each of the species in this study (such as *ampC* in *E. coli* and *shv* in *K. pneumoniae*), and little of the functional consequence of this diversity is understood. In general, the chromosomal *ampC*'s of *E. coli* differ from plasmid-borne class C β-lactamases and the chromosomal β-lactamases of *K. pneumoniae* or *Enterobacter* species in that *E. coli ampC*'s do not provide resistance against cephalosporins. This is thought to be primarily a result of promoter strength, but it leaves open the question of whether all *ampC* genes can be treated the same in GBASP or not. As a greater diversity of *ampC* genes and promoters are studied, a set of rules may be identified that define the functional effect of specific mutations, allowing us to treat the chromosomal *ampC* β-lactmases similarly to how we treated *gyrA* or *parC* genes for fluoroquinolone resistance. Alternatively, if sufficient *E. coli* isolates were phenotyped and sequenced, the results could be used through machine learning to build a classification model capable of distinguishing the various *ampC* genes observed in pathogenic *E. coli*.

The third challenge is technical: draft genome sequences are more cost efficient to produce than complete genomes, but they may have assembly breaks within key genes, leading to prediction errors. The impact of this limitation was most obvious in our ciprofloxacin predictions, where incompletely assembled QRDR regions resulted in predictions that were not determined (Figure 3). This type of error was more difficult to identify when the incompletely assembled gene was part of the accessory genome but we anticipate that some of our very major errors in each antibiotic condition resulted from incomplete genome assembly. Long-read sequence technologies, such as SMRT sequencing (http://www.pacb.com) and nanopore sequencing (nanoporetech.com) hold promise for generating finished genomes in a single run, potentially reducing this source of error for future datasets.

The final challenge is the choice of gold standard technique by which to evaluate and refine predictive algorithms. We chose to compare to disc-diffusion, because it was the technique being used in the hospital from which the isolates were taken. In addition, the discrete interpretation of disk diffusion results (susceptible, intermediate, or resistant) is amenable to comparison to GBASP. The accepted, inherent error in any *in vitro* susceptibility testing method is a variance of plus or minus one doubling dilution for the minimum inhibitory concentration value. While we cannot evaluate the complete effect that the variability inherent in phenotype-based AST has on our analysis, our repeat analysis of the doxycycline testing showed that some of the disagreement between our predictions and the *in vitro* susceptibility testing were due to variable interpretations of results for isolates near the border between susceptible and non-susceptible. This may have been particularly true for the doxycycline predictions because of the large number of isolates near that border, but we hypothesize that it may have had some effect on all of our predictions. A 4% increase in agreement between the LR algorithm and the gold standard (such as was seen for Doxycycline in our limited repeat testing) would have led to a greater than 95% accuracy for 8 of the 12 antibiotics tested. For this reason, we advocate the use of robust gold standard data for future algorithm testing.

Our results indicate that GBASP has the potential to move beyond an early-results supplement to phenotypic AST, and may, in the near future, be able to fully replace methods based on isolate growth. This can be a gradual process; GBASP can be implemented to provide early treatment suggestions before more definitive answers are available from phenotypic AST, similar to how current versions of genotypic diagnostics are used. As GBASP improves, due to larger training sets and more concrete knowledge of the connections between resistance genotypes and phenotypes, it can be periodically evaluated for its reliability. While this process will require concerted effort, the benefits of informed initial antibiotic treatments make developing GBASP a key global public health imperative.

## Author contributions

TH performed microbiology and DNA isolation. TH and SP performed functional metagenomics. MW performed disc diffusion assays. MP performed sequence analysis, GBASP algorithm implementation, and data visualization. MP, TH, SA, CB, and GD designed the study. MP, CB, and GD wrote the manuscript.

## Funding

Research reported in this publication was supported in part by the NIH Director's New Innovator Award (http://commonfund.nih.gov/newinnovator/), the National Institute of Diabetes and Digestive and Kidney Diseases (NIDDK: http://www.niddk.nih.gov/), and the National Institute of General Medical Sciences (NIGMS: http://www.nigms.nih.gov/), of the National Institutes of Health (NIH) under award numbers DP2DK098089 and R01GM099538 to GD. The content is solely the responsibility of the authors and does not necessarily represent the official views of the NIH. MP is supported by the NIGMS Cell and Molecular Biology Training Grant (GM: 007067). TH is supported by the Higher Education Commission of Pakistan's International Research Support Initiative Program.

### Conflict of interest statement

The authors declare that the research was conducted in the absence of any commercial or financial relationships that could be construed as a potential conflict of interest.
